# A Solar Altitude Angle Model for Efficient Solar Energy Predictions

**DOI:** 10.3390/s20051391

**Published:** 2020-03-04

**Authors:** Sergio Herrería-Alonso, Andrés Suárez-González, Miguel Rodríguez-Pérez, Raúl F. Rodríguez-Rubio, Cándido López-García

**Affiliations:** Department of Telematics Engineering, University of Vigo, 36310 Vigo, Spain; asuarez@det.uvigo.es (A.S.-G.); miguel@det.uvigo.gal (M.R.-P.); rrubio@det.uvigo.es (R.F.R.-R.); candido@det.uvigo.es (C.L.-G.)

**Keywords:** energy harvesting, solar energy, energy management, energy prediction

## Abstract

Sunlight is one of the most frequently used ambient energy sources for energy harvesting in wireless sensor networks. Although virtually unlimited, solar radiation experiences significant variations depending on the weather, the season, and the time of day, so solar-powered nodes commonly employ solar prediction models to effectively adapt their energy demands to harvesting dynamics. We present in this paper a novel energy prediction model that makes use of the altitude angle of the sun at different times of day to predict future solar energy availability. Unlike most of the state-of-the-art predictors that use past energy observations to make predictions, our model does not require one to maintain local energy harvesting patterns of past days. Performance evaluation shows that our scheme is able to provide accurate predictions for arbitrary forecasting horizons by performing just a few low complexity operations. Moreover, our proposal is extremely simple to set up since it does not require any particular tuning for each different scenario or location.

## 1. Introduction

Energy harvesting (EH) is the process that enables the conversion of ambient energy present in the environment (e.g., solar, wind, thermal, or kinetic energy) into electrical energy for use in powering small, autonomous devices. This promising technology encourages the deployment of long-term wireless networks since conventional, battery-operated devices can be replaced by self-sustainable, autonomous ones that harvest the energy they need from their surrounding environment [1]. The quest for perpetually communicating devices is challenged by the intermittent and random nature of ambient energy sources. For example, energy harvested from sunlight or wind experiences significant variations depending on the weather, the season, and the time of day. For this reason, part of the scavenged energy is usually stored in a component of limited capacity (typically, a supercapacitor or a battery) to further balance the amount of energy harvested at each moment with the energy consumption profile.

EH technology can be used to provide a virtually uninterrupted power supply to the nodes of a Wireless Sensor Network (WSN) [2]. However, to cope with the uncertainty in the energy availability, EH nodes must implement an energy management policy to ensure long-term operations [3]. This management policy determines when to activate/deactivate the sensing, transmitting, or receiving circuits of the device according to the amount of available energy; i.e., it tries to schedule sensing and communication tasks optimally from an energy point of view. Certainly, the design of optimal management policies requires the energy harvesting profile to be known in advance or, at least, that it can be accurately predicted [4,5].

In energy harvesting WSNs (EH-WSNs), accurate prediction of the near future energy availability (from a few minutes to a few hours) is particularly important to avoid short-term energy shortages at the sensor nodes [6]. In this paper, we focus on one of the most common and effective ambient energy sources for EH-WSNs: sunlight. Solar radiation is dynamic, uncontrollable, and only partially predictable. The solar energy available to a device can fluctuate considerably even within a short period. There is a need, therefore, for efficient prediction methods that accurately forecast the expected solar energy intake in the near future so that energy expenditure can be effectively adapted to the dynamics of energy harvesting.

Different estimation techniques have been proposed in the last few years. Most of them use past energy patterns to predict future energy availability, thus requiring one to maintain several profiles of the energy harvested by the EH node for a number of prior days [6,7,8,9,10,11,12,13,14,15,16]. In this paper, we present a novel solar energy model, named the Solar Altitude Angle (SAA) model, that adopts a completely different approach. It is well known that solar irradiance on a cell surface mainly depends on the angle of incidence of the sunlight on it, that is, on the height of the sun above the horizon. Hence, the SAA model only uses the solar altitude angles at different times of day to predict future solar energy intake. Evaluation results using real datasets show that our proposal is able to provide accurate predictions at both short-term and medium-term forecasting horizons by only executing a few low complexity operations. In addition, since it does not maintain locally collected past energy measures, the SAA model has a very low memory overhead. Finally, it is important to highlight that our proposal is extremely simple to set up since, unlike most prediction schemes, it does not require the careful tuning of any configuration parameter to ensure accurate energy estimations. In fact, the SAA model can be used without requiring any particular configuration in different solar energy harvesters under varying weather conditions and locations. All these features make our proposal particularly suitable for hardware constrained devices (for example, sensor-like motes) that must work under severe resource restrictions such as limited battery and computing power, and/or scarce memory [17].

The rest of the paper is organized as follows. Related work is reviewed in Section 2. In this section, we also briefly describe Pro-Energy and UD-WCMA predictors, two of the most performant schemes. Section 3 presents the SAA model, our novel solar energy model. In Section 4, we evaluate the performance of the SAA model and compare it with that of Pro-Energy and UD-WCMA predictors in different scenarios. In Section 5, we discuss some implementation issues. The main conclusions are laid out in Section 6.

## 2. Related Work

Many different prediction models have been proposed in recent years. Most of them use energy observations in prior days to predict future energy availability: QL-SEP [6], EWMA [7], WCMA [8], ASEA [9], Pro-Energy [10,11], IPro-Energy [12], SEPCS [13], UD-WCMA [14], LINE-P [15], and Adaptive LINE-P [16]. This class of models requires maintaining locally collected data about the energy harvested during a number of prior days. Subsequently, recently measured energy values are carefully combined with the energy harvesting patterns from previous days to predict future energy intake. For example, EWMA [7] estimates the energy value at timeslot *n* as a weighted average of the most recent energy value and the mean energy value at timeslot *n* of the previous days. QL-SEP [6] adjusts the EWMA estimation with the average increase/decrease ratio (either positive or negative) of the energy harvested in the previous timeslots. ASEA [9] improves the EWMA model introducing an additional scaling factor that adjusts future energy expectations based on the variability of short-term weather conditions. WCMA [8] also improves EWMA in scenarios with changing weather conditions. This model introduces a new weighting factor that quantifies how many current weather conditions have changed with respect to those in the previous days. UD-WCMA [14] is a dynamic version of WCMA with adaptive weighting factors. Pro-Energy [11] carefully combines the most recent energy value with the average energy harvested during the most similar previous days to the current one. We will provide a more detailed description of Pro-Energy and UD-WCMA in the following subsections. SEPCS [13] and LINE-P [15] employ complex linear prediction models that take into account both previous energy samples from the same day and energy samples from previous days. Finally, Adaptive LINE-P [16] improves LINE-P with adaptive weighting parameters.

All of these techniques rely on one or several weighting factors that determine the relative importance between current and past measures when computing energy predictions. As expected, the configuration of these parameters is critical for accurate predictions. Moreover, there is no single set of parameter values that performs well enough for all weather conditions and/or energy harvesters. Generally, the weighting factors are tuned *a priori* for a particular set of data, so most of these techniques cannot dynamically adapt to varying weather conditions. To the best of our knowledge, only UD-WCMA [14] and Adaptive LINE-P [16] predictors provide effective models able to dynamically adjust the weighting factors to current weather conditions.

There is also another class of models that use cloud cover forecasts to predict solar energy intake [18,19]. These methods are able to improve long-term predictions but can only be applied to scenarios in which external connectivity is available to obtain weather forecasts. In this paper, we focus on those schemes that only use locally collected energy measures to make predictions, as they are suitable for autonomous EH nodes with no online resources available. Among them, two of the most popular schemes are the Pro-Energy and UD-WCMA predictors. We briefly present both prediction methods in the following subsections.

### 2.1. Pro-Energy

This predictor divides each day into *N* non-overlapping timeslots of equal duration. To make predictions, Pro-Energy maintains a pool of *D* profiles, each one containing the energy harvested during each of the *N* timeslots of a given past day. Subsequently, at timeslot *n* of the current day C, the energy prediction for future timeslot n+i, i∈{1,2,…}, is computed as follows:

(1) $${\hat{E}}_{n+i}^{C}=\gamma_{i}\;E_{n}^{C}+\left(1-\gamma_{i}\right)\;{\bar{E}}_{n+i}^{P}\;,$$

where ${\hat{E}}_{n+i}^{C}$ is the energy predicted for timeslot n+i of the current day, $E_{n}^{C}$ is the energy harvested during the last timeslot *n* of the current day, ${\bar{E}}_{n+i}^{P}$ is the average energy harvested during timeslot n+i of the *P* most similar profiles of the pool, and $\gamma_{i}$ is the correlation factor that determines the significance of the last measured energy value $E_{n}^{C}$ when computing predictions for future timeslots:

(2) $$\gamma_{i}={\left\{\alpha \left(1-\frac{i-1}{G}\right)\right\}}^{+},$$

with α a weighting factor, 0≤α≤1, and *G* the number of timeslots in the future that presumably show a strong correlation with the energy harvested during the current timeslot. Clearly, the weight associated with the last measured energy value progressively decreases as predictions go away in time.

To compute ${\bar{E}}_{n+i}^{P}$, the similarity with the current day for each profile $\left\{p_{1},p_{2},\cdots ,p_{D}\right\}$ of the pool must be computed. For a given profile p, this will be computed as the mean absolute error (MAE) over the previous *K* timeslots:

(3) $${\mathrm{MAE}}_{K}\left(p,C\right)=\sum_{j=n-K+1}^{n}\frac{1}{K}\left|E_{j}^{C}-E_{j}^{p}\right|\;,$$

where $E_{j}^{p}$ is the energy harvested during timeslot *j* of profile p. Subsequently, if we assume that $\left\{p_{1},p_{2},\cdots ,p_{D}\right\}$ is the ordered set of profiles based on their similarity with the current day, the weighted average energy value is computed as

(4) $${\bar{E}}_{n+i}^{P}=\left\{\begin{array}{cc} E_{n+i}^{p_{1}}\;, & \;\mathrm{if}\;P=1,\\ \frac{1}{P-1}\sum_{j=1}^{P}w_{j}\;E_{n+i}^{p_{j}}\;, & \;\mathrm{if}\;P>1, \end{array}\right.$$

where P<D is the number of profiles combined, and

(5) $$w_{j}=1-\frac{{\mathrm{MAE}}_{K}\left(p_{j},C\right)}{\sum_{q=1}^{P}{\mathrm{MAE}}_{K}\left(p_{q},C\right)}\;.$$

### 2.2. Dynamic Weather Condition Moving Average (D-WCMA)

D-WCMA predictions are based on a weighting factor that is dynamically configured to adapt to different weather conditions. This model needs to store the energy harvested in each of the *N* timeslots of the *D* previous days $\left\{d_{1},d_{2},\ldots ,d_{D}\right\}$. Subsequently, at timeslot *n* of the current day C, D-WCMA computes the energy prediction for future timeslot n+i as follows:

(6) $${\hat{E}}_{n+i}^{C}=\alpha_{n+i}\;E_{n}^{C}+\left(1-\alpha_{n+i}\right)\;{\mathrm{GAP}}_{K}\;{\bar{E}}_{n+i}^{D}\;,$$

where $\alpha_{n+i}$ is the adaptive weight of the last observation in the prediction for timeslot n+i, GAP${}_{K}$ is a factor that scales the current energy variations with respect to those of the previous days over a time horizon of *K* timeslots, and ${\bar{E}}_{n+i}^{D}$ is the average energy harvested during timeslot n+i in the previous *D* days:

(7) $${\bar{E}}_{n+i}^{D}=\frac{1}{D}\sum_{j=1}^{D}E_{n+i}^{d_{j}}\;,$$

where $E_{n+i}^{d_{j}}$ is the energy harvested during timeslot n+i of the preceding day $d_{j}$.

The weighting factor $\alpha_{n+i}$ estimates the predictability level of the energy intake during future timeslot n+i from the variations in the energy harvested during the previous days. It is dynamically configured as follows:

(8) $$\alpha_{n+i}=\frac{1}{2}\frac{\sigma_{n+i}}{\sigma_{n+i}+\sigma_{n+i}^{\prime }}\;,$$

where

(9) $$\begin{array}{cc} \sigma_{n+i} & =\sqrt{\frac{1}{D}\sum_{j=1}^{D}{\left(E_{n+i}^{d_{j}}-{\bar{E}}_{n+i}^{D}\right)}^{2}}\;,\\ \sigma_{n+i}^{\prime } & =\sqrt{\frac{1}{D}\sum_{j=1}^{D}{\left(∆E_{n+i}^{d_{j}}-{\bar{∆E}}_{n+i}^{D}\right)}^{2}}\;,\\ ∆E_{n+i}^{d_{j}} & =E_{n+i}^{d_{j}}-E_{n}^{d_{j}}\;,\\ {\bar{∆E}}_{n+i}^{D} & =\frac{1}{D}\sum_{j=1}^{D}∆E_{n+i}^{d_{j}}\;. \end{array}$$

$\sigma_{n+i}$ is the standard deviation of the energy harvested during timeslot n+i of the preceding days, whereas $\sigma_{n+i}^{\prime }$ is the standard deviation of the energy variations between timeslots *n* and n+i on those days. Finally, the GAP factor is computed as a normalized weighted average of the ratio between the energy harvested in the current day and the average energy harvested in the previous days along the last *K* timeslots:

(10) $${\mathrm{GAP}}_{K}=\frac{\sum_{k=1}^{K}\frac{k}{K}\frac{E_{n-K+k}^{C}}{{\bar{E}}_{n-K+k}^{D}}}{\sum_{k=1}^{K}\frac{k}{K}}=\frac{2}{K(K+1)}\sum_{k=1}^{K}k\;\frac{E_{n-K+k}^{C}}{{\bar{E}}_{n-K+k}^{D}}\;.$$

### 2.3. Universal Dynamic WCMA (UD-WCMA)

For the purpose of improving robustness to weather variations, UD-WCMA replaces in the D-WCMA prediction scheme the last measured energy value, $E_{n}^{C}$, with a weighted linear combination of itself and the energy harvested during the future timeslot in the most similar previous day. Thus, the energy prediction for future timeslot n+i is computed as follows:

(11) $${\hat{E}}_{n+i}^{C}=\alpha_{n+i}\left(\beta_{n+i}\;E_{n}^{C}+\left(1-\beta_{n+i}\right)E_{n+i}^{d^{*}}\right)+\left(1-\alpha_{n+i}\right)\;{\mathrm{GAP}}_{K}\;{\bar{E}}_{n+i}^{D}\;,$$

where $\beta_{n+i}$ is the new weighting factor used in the prediction for timeslot n+i, and $E_{n+i}^{d^{*}}$ is the energy harvested during timeslot n+i of day $d^{*}$, $d^{*}$ being the day among the *D* previous days with the more similar energy profile to the current day. The similarity between days is computed in the same way as in the Pro-Energy predictor. The weighting factor β is dynamically tuned as follows:

(12) $$\beta_{n+i}=\alpha_{n+i}+\frac{1}{2}\frac{\sigma_{n+i}}{\sigma_{n+i}+\sigma_{n}^{\prime \prime }}\;,$$

where $\sigma_{n}^{\prime \prime }$ is the standard deviation of the variations in the energy harvested in the current day along the last K−1 consecutive timeslots:

(13) $$\begin{array}{cc} \sigma_{n}^{\prime \prime } & =\sqrt{\frac{1}{K-1}\sum_{k=0}^{K-2}{\left(∆E_{n-k}^{C}-{\bar{∆E}}_{n}^{K}\right)}^{2}}\;,\\ ∆E_{n-k}^{C} & =E_{n-k}^{C}-E_{n-k-1}^{C}\;,\;k=0,\ldots ,K-2\;,\\ {\bar{∆E}}_{n}^{K} & =\frac{1}{K-1}\sum_{k=0}^{K-2}∆E_{n-k}^{C}\;. \end{array}$$

## 3. Solar Altitude Angle Energy Predictor

**Figure 1:**
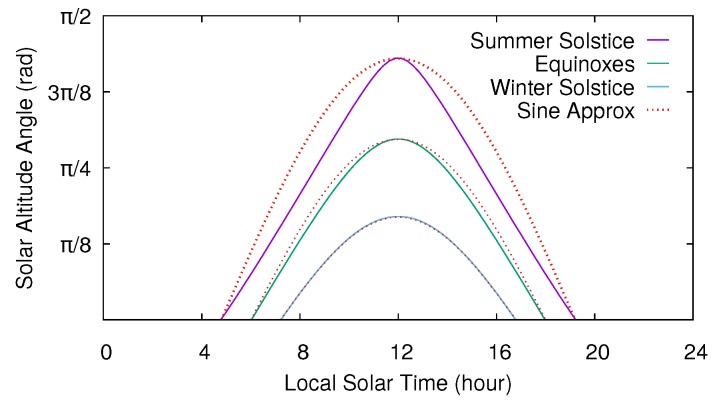
Altitude angle of the sun through the course of various days for an observer at 36°N latitude. Sine approximations are shown with dotted lines.

Among other factors (atmospheric effects, cloud cover, air pollution, and so on), solar irradiance on the surface of a solar cell depends on the angle of incidence of the sunlight on it and, therefore, on the height of the sun above the horizon. The altitude angle (or elevation angle) is the angular height of the sun in the sky measured from the horizon. The altitude angle varies throughout the day and depends on the observer’s latitude and the day of year (see Figure 1). Certainly, the altitude angle is zero at sunrise and sunset times, and solar irradiance is negligible between the sunset of a day and the sunrise of the next day. From sunrise, the altitude angle (and, therefore, solar irradiance in the absence of changes in atmospheric conditions) increases following, approximately, a sine curve. The maximum solar irradiance is achieved at noon, the time when the sun apparently reaches its highest point in the sky. Finally, from noon to sunset, the altitude angle (and thus the solar irradiance) decreases in a symmetrical way.

### 3.1. Solar Altitude Angle Computation

The altitude angle θ depends on the observer’s latitude, time, and day of year, and it can be computed using the formula:

(14) $$\theta =\arcsin\left(\sin\delta \sin\varphi +\cos\delta \cos\varphi \cos\omega \right),$$

where δ is the declination angle, φ is the latitude angle, and ω is the hour angle [20].

The declination angle is the angle between the equator and a line drawn from the center of the Earth to the center of the sun. This angle can be easily computed using the well-known approximation

(15) $$\delta =-0.40928\cos\left(\frac{2\pi }{365}\;\left(d+10\right)\right),$$

where *d* is the day of year (such that d=1 on 1 January, and so on) [21]. Note that, by definition, the declination angle is independent of the observer’s position on the Earth’s surface and only depends on the day of year.

The hour angle converts the local solar time into the amount of radians the sun moves across the sky. Note that, by convention, the hour angle is zero at solar noon, and, since the Earth rotates 2π/24 rad per hour, the hour angle ω can be simply calculated as follows:

(16) $$\omega =\frac{\pi }{12}\;\left(t-12\right)\;,$$

where *t* is the local solar time in hours.

### 3.2. Solar Altitude Angle Energy Prediction

Instead of inspecting past energy observations, we suggest making use of the altitude angle of the sun to predict future solar energy intake. Thus, at timeslot *n*, the energy harvested for a future timeslot n+i of the current day C, ${\hat{E}}_{n+i}^{C}$, can be estimated by linearly interpolating the most recent known energy value, that is, the energy harvested during the last timeslot, $E_{n}^{C}$, from the altitude angles of the sun at the times corresponding to the given timeslots:

(17) $${\hat{E}}_{n+i}^{C}=E_{n}^{C}\;\frac{\;\theta_{n+i}\;}{\;\theta_{n}\;}\;,$$

where $\theta_{n}$ and $\theta_{n+i}$ are, respectively, the solar altitude angles at times $t_{n}$ and $t_{n+i}$, that is, the times corresponding to timeslots *n* and n+i of the current day. Certainly, this model is assuming that atmospheric and weather conditions at $t_{n+i}$ remain similar to those at $t_{n}$, but this is actually the most likely scenario for short-term prediction horizons.

### 3.3. Solar Altitude Angle Sine Approximation

As we just explained, the estimation of the solar altitude angle requires the computation of the declination angle, the hour angle, and several trigonometric functions. Although these operations are not excessively complex, the computation of the altitude angle can be simplified if it is approximated by only a sine curve. Since the altitude angle is maximum at noon, and zero at sunrise and sunset, it can be approximated between sunrise and sunset times by the following sine function:

(18) $$\theta_{t}\approx \theta_{\mathrm{noon}}\sin\left(\pi \frac{t-t_{\mathrm{rise}}}{t_{\mathrm{set}}-t_{\mathrm{rise}}}\right),\;t_{\mathrm{rise}}\leq t\leq t_{\mathrm{set}}\;,$$

where $\theta_{\mathrm{noon}}$ is the solar altitude angle at noon (the maximum altitude angle), and $t_{\mathrm{rise}}$ and $t_{\mathrm{set}}$ are, respectively, the sunrise and sunset times of the given day. Figure 1 shows both the exact altitude angle and the approximated value using Equation (18) through the course of several representative days. As shown, the sine approximation provides very accurate values for the days with the lowest amount of hours of sunlight of the year. On the contrary, for those days with the most sunlight, this approximation is less precise, though it still obtains acceptable values since it follows a curve that is similar to the real one, and we actually use the ratio between the angles at the current time and the prediction horizon to compute the energy estimations. In any case, from Equation (17) and using the sine approximation expressed in Equation (18), we can estimate at timeslot *n* the energy harvested for a future timeslot n+i of the current day C as

(19) $${\hat{E}}_{n+i}^{C}=E_{n}^{C}\;\frac{\;\sin\left(\pi \left(t_{n+i}-t_{\mathrm{rise}}\right)/\left(t_{\mathrm{set}}-t_{\mathrm{rise}}\right)\right)}{\sin\left(\pi \left(t_{n}-t_{\mathrm{rise}}\right)/\left(t_{\mathrm{set}}-t_{\mathrm{rise}}\right)\right)}\;.$$

### 3.4. Solar Incidence Angle on a Tilted Surface

**Figure 2:**
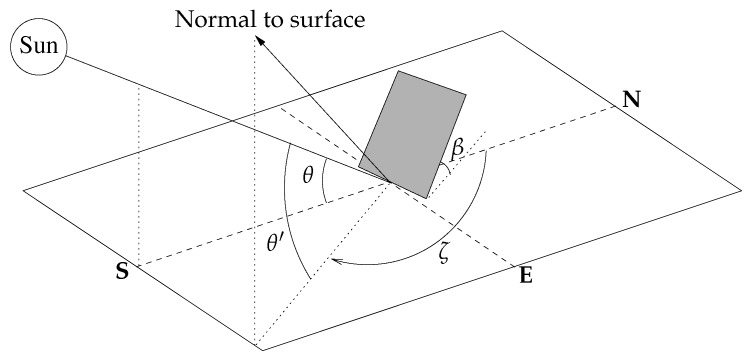
Solar incidence angle on a tilted surface.

Clearly, the angle of incidence of the sun on a horizontal surface coincides with the solar altitude angle. However, the angle of incidence varies in the case of a tilted surface (see Figure 2). The solar incidence angle on a surface tilted at an angle β from the horizontal can be calculated using the following formula:

(20) $$\begin{array}{cc} \theta^{\prime }=\arcsin\;( & \sin\delta \sin\varphi \cos\beta +\sin\delta \cos\varphi \sin\beta \cos\zeta +\cos\delta \cos\varphi \cos\omega \cos\beta \\ \; & -\cos\delta \sin\varphi \cos\omega \sin\beta \cos\zeta -\cos\delta \sin\omega \sin\beta \sin\zeta )\;, \end{array}$$

where ζ is the surface azimuth angle, i.e., the angle between the normal to the surface from north (clockwise measured). Note that, for horizontal surfaces (β=0), Equation (20) reduces to Equation (14) and $\theta^{\prime }=\theta$.

**Figure 3:**
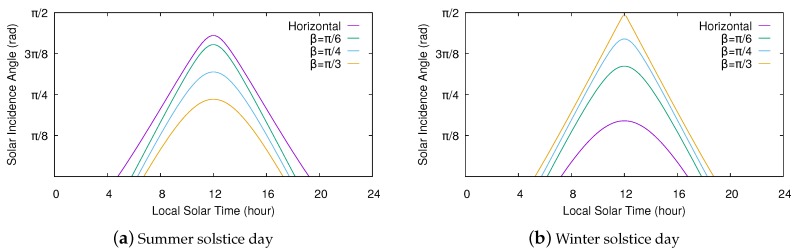
Solar incidence angle through the course of solstice days for a tilted surface facing the equator (ζ=π) at 36°N latitude.

Usually, solar cells are aligned to face the equator. Thus, solar cells in the northern hemisphere will typically face directly south (ζ=π), while those in the southern hemisphere will face directly north (ζ=0). Figure 3 shows how the solar incidence angle varies on a tilted surface facing the equator at different tilt angles. Note that, through the course of the summer solstice day, the incidence angle at a particular time decreases as β increases. Contrarily, the incidence angle increases with β during the winter solstice day. Consequently, steeper tilt angles are preferred during the winter in the northern hemisphere, whereas lower tilt angles enable solar cells to capture more sunlight during the summer. In any case, although the surface slant clearly modifies the solar incidence angle, this consistently follows a curve similar to that obtained for a horizontal surface, so our energy model should perform well even for those tilted cells with an unknown tilt angle.

## 4. Evaluation

**Figure 4:**
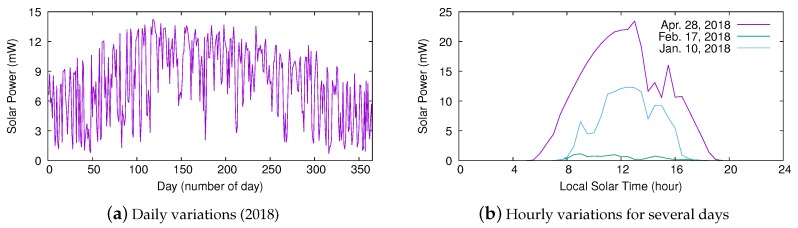
Solar power harvesting variations at Oak Ridge National Laboratory.

We evaluated the performance of Pro-Energy, UD-WCMA, and Solar Altitude Angle (SAA) predictors using an open-source in-house simulator [22]. We employed real solar traces obtained from the National Renewable Energy Laboratory (NREL) at Oak Ridge, Tennessee [23]. Each original trace contains one sample per minute of the radiant energy on the solar cell in a given day. From the raw irradiance values *I*, the harvested power has been estimated as A·η·I, where η is the efficiency factor of the solar cell, and *A* is the area of the cell surface. As in [11], we considered η=0.17 and A= 22 mm×7mm. In addition, we set the number of timeslots per day *N* to 48, so we stored in each energy profile the average solar power harvested at each 30 min interval. Figure 4 depicts the average power harvested from sunrise to sunset for each day of the year 2018 and the power harvested at different times of several representative days for the given location. Note how solar power harvesting significantly varies from one day to another and at different times of the same day.

We evaluated the accuracy of the prediction methods for timeslot horizons (*h*) from 1 to 4 (30 min to 120 min). We computed the predictions for each day of the year 2018 from sunrise to sunset and, afterward, the MAE and the Mean Absolute Deviation (MAD) for all of them:

(21) $$\begin{array}{cc} \mathrm{MAE} & =\frac{\sum |E_{\left[n,n+h\right]}-{\hat{E}}_{\left[n,n+h\right]}|}{\;\mathrm{number}\;\mathrm{of}\;\mathrm{predictions}\;}\;,\\ \mathrm{MAD} & =\frac{\sum |E_{\left[n,n+h\right]}-{\hat{E}}_{\left[n,n+h\right]}|}{\sum E_{\left[n,n+h\right]}}\;, \end{array}$$

where $E_{\left[n,n+h\right]}$ and ${\hat{E}}_{\left[n,n+h\right]}$ are, respectively, the actual and the predicted solar energy harvested from timeslot *n* to timeslot n+h:

(22) $$E_{\left[n,n+h\right]}=\sum_{i=n+1}^{n+h}E_{i}^{C}\;\;\mathrm{and}\;\;{\hat{E}}_{\left[n,n+h\right]}=\sum_{i=1}^{h}{\hat{E}}_{n+i}^{C}.$$

### 4.1. Optimal Pro-Energy Configuration

We firstly evaluated the performance of the Pro-Energy predictor with multiple configuration settings. Note that, contrary to our proposal, the computational overhead introduced by this scheme (and, consequently, the accuracy of its predictions) depends on the setting of several parameters such as the number of energy profiles stored (*D*), the number of past measures used to compute similarity between profiles (*K*), and the number of profiles combined for predictions (*P*). We configured this predictor with three different sets of parameters (overhead levels), as shown in Table 1. Additionally, to compute the correlation factor γ, we always used G=5 (2.5h), while the weighting factor α was configured with values from 0 to 1 to find out the best value for each simulated scenario.

**Figure 5:**
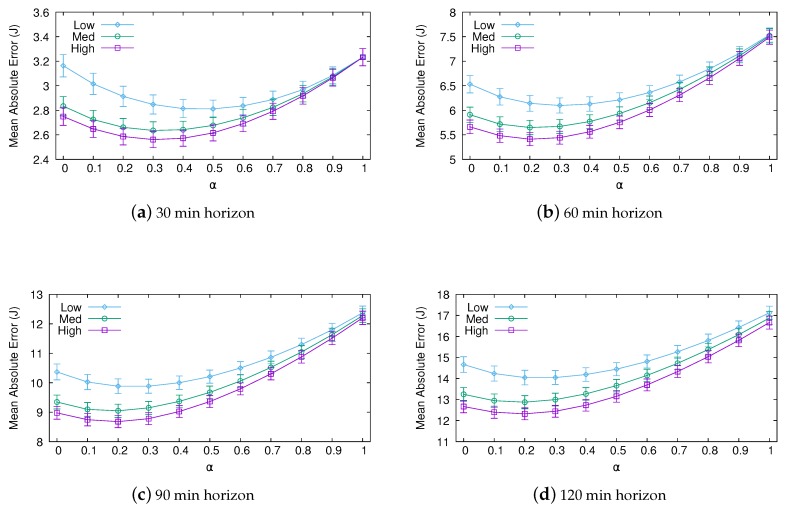
Mean absolute error (MAE) of short-term and medium-term predictions obtained with Pro-Energy.

Figure 5 shows the MAE for both the short-term (30 and 60 min) and medium-term (90 and 120 min) predictions obtained with Pro-Energy (95% confidence intervals are shown in the graphs). As expected, prediction errors are greater as the prediction horizon moves away, and the most accurate predictions are achieved using the setting with the highest overhead. Note that the α parameter greatly impacts performance. The optimal weighting factor takes values from 0.5 for the 30 min predictions with the low overhead settings to 0.2 for the 120 min predictions with the high overhead settings. Not surprisingly, the relative significance of the last measured energy value (that is, the weighting factor α) must be diminished as the prediction horizon moves away.

### 4.2. Performance Comparison

We compared the performance of the SAA energy predictor with respect to the Pro-Energy and UD-WCMA schemes. To ensure a fair comparison, we configured Pro-Energy with the optimal α parameter in each scenario, as obtained in the previous section. For UD-WCMA, we set D=6 and K=4 as recommended in [14]. Recall that our predictor does not require the tuning of any parameter at all.

**Figure 6:**
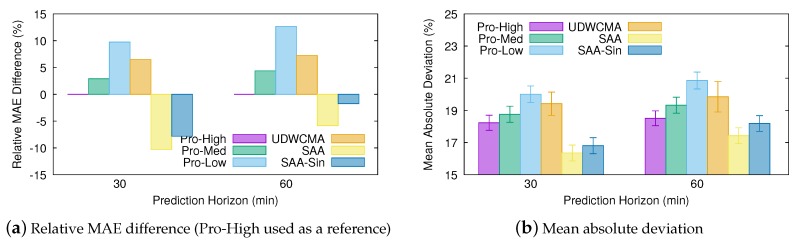
Short-term prediction error comparison.

Figure 6 shows the short-term prediction results obtained with the different estimation methods—in particular, their MAD—and the relative difference between the MAE obtained with each of the predictors and that obtained with Pro-Energy when configured with the high overhead settings (Pro-High), since this is the best performing alternative. Noticeably, and despite its apparent simplicity, the MAE obtained with the SAA model is 10.3% and 5.9% lower than that obtained with Pro-High at 30 and 60 min prediction horizons, respectively. Moreover, even the simplified SAA model using the sine approximation (SAA-Sine) reduces the MAE obtained with Pro-High by 7.8% and 1.8% at 30 and 60 min horizons, respectively. On the other hand, the UD-WCMA technique is able to slightly improve the predictions given by Pro-Energy with the low overhead settings (Pro-Low) only, but its estimations are much less accurate than those obtained with our proposal.

**Figure 7:**
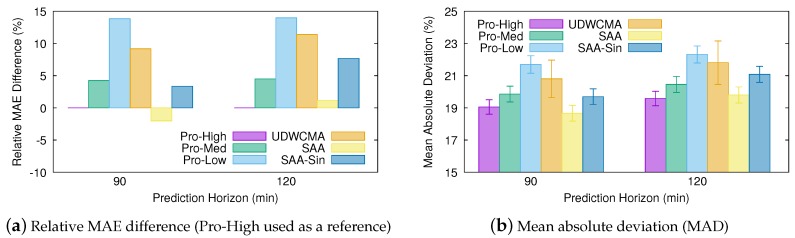
Medium-term prediction error comparison.

The results obtained for the medium-term predictions are shown in Figure 7. Pro-High provides slightly more accurate estimations than the SAA technique only at the 120 min prediction horizon. Moreover, even SAA-Sine still achieves more precise estimations than both UD-WCMA and Pro-Low. Consequently, our SAA model would be a very interesting choice for solar energy forecasting at both short- and medium-term prediction horizons.

## 5. Implementation

In this section, we will evince that our model presents clear advantages for implementation in hardware-constrained devices. Recall that the SAA predictor does not use energy harvesting patterns observed in the past to estimate future energy availability. Instead, it just requires the computation of the altitude angle of the sun at both the current time and the future prediction horizon. Fortunately, this is not an arduous task. As shown in Section 3, the solar altitude angle can be computed using Equation (14) from the declination, the hour, and the latitude angles. Note first that stationary nodes can be preloaded with the sine and cosine of their corresponding latitude angle. Furthermore, recall from Equation (15) that the solar declination angle only depends on the day of year, so this angle can only take 365 different values that could be easily pre-calculated and stored in the nodes. In fact, the corresponding sines and cosines of the declination angles could be directly stored in the nodes. If this were not the case, the declination angle should be computed daily. In any case, a variable storing the current day of year is necessary and must be incremented (modulo-365) by one once a day.

To compute the declination angle (and its corresponding sine and cosine), we must perform some trigonometric functions. To estimate their complexity, we assume that they are approximated using a finite number of terms of their corresponding Taylor series. Thus, the sine and cosine functions for a given angle of *x* rad can be calculated as follows:

(23) $$\begin{array}{cc} \sin x & =\sum_{n=0}^{\infty }\frac{{\left(-1\right)}^{n}}{(2n+1)!}x^{2n+1}=x-\frac{x^{3}}{3!}+\frac{x^{5}}{5!}-\frac{x^{7}}{7!}+\ldots \\ & =x(1-x^{2}(0.166667-x^{2}(0.008333-x^{2}(0.000198-\ldots \end{array}$$

(24) $$\begin{array}{cc} \cos x & =\sum_{n=0}^{\infty }\frac{{\left(-1\right)}^{n}}{(2n)!}x^{2n}=1-\frac{x^{2}}{2!}+\frac{x^{4}}{4!}-\frac{x^{6}}{6!}+\ldots \\ & =1-x^{2}(0.5-x^{2}(0.041667-x^{2}(0.001389-\ldots \end{array}$$

The 7-th (8-th) degree Taylor polynomial provides a good estimation of the sine (cosine) function in the range [−π:π], so we assume that computing each sine (cosine) function involves performing 3 additions and 5 multiplications (4 additions and 5 multiplications). This is a worst case scenario since, for small angles, these functions can be accurately approximated with a Taylor polynomial of a lower degree. Subsequently, it easily follows from Equation (15) that computing the declination angle requires a total of 5 additions and 7 multiplications and, since what we need to estimate the altitude angle is its sine and cosine, we have to perform 7 more additions and 10 more multiplications. The product of the sines (and cosines) of the latitude and the declination angles needed to compute the altitude angle also only has to be calculated once a day.

Contrastingly, the hour angle (and its cosine) and the altitude angle at the prediction horizon must be computed for each prediction. Note that the altitude angle at the current timeslot will have already been computed in the previous prediction (as the altitude angle at the prediction horizon), so it is unnecessary to calculate it again. The hour angle can be easily calculated using Equation (16), so only one addition and one multiplication is required. The arcsine function needed to compute the altitude angle can be calculated as follows:

(25) $$\begin{array}{cc} \arcsin x & =\sum_{n=0}^{\infty }\frac{(2n)!}{4^{n}{\left(n!\right)}^{2}\left(2n+1\right)}x^{2n+1}=x+\frac{1}{6}x^{3}+\frac{3}{40}x^{5}+\frac{15}{336}x^{7}+\ldots \\ \; & =x(1+x^{2}(0.166667+x^{2}(0.075+x^{2}(0.044643+\ldots , \end{array}$$

thus requiring the same number of operations as the sine function. Finally, only one extra multiplication and division must be performed to estimate the future energy intake (see Equation (17)).

In the case of using the sine approximation expressed in Equation (19), both sunrise and sunset times of the current day at the given location must be known beforehand. These times can be directly estimated from previous measures. For example, the sunrise time ($t_{\mathrm{rise}}$) can be estimated as the time corresponding to the first timeslot of the current day in which the amount of harvested solar energy is not null (or above a minimum threshold). Similarly, the current day length, that is, the interval from sunrise to sunset ($t_{\mathrm{set}}-t_{\mathrm{rise}}$), can be approximated as the day length observed in the previous day, since day length only varies a few minutes per day. These times can be measured in any unit, even in the number of timeslots directly. Subsequently, to make a prediction, the sine of the relative angle corresponding to the future timeslot must be computed. Recall that the sine of the relative angle at the current timeslot will have already been computed in the previous prediction (as the sine value for the future timeslot). This angle includes the factor $\pi /(t_{\mathrm{set}}-t_{\mathrm{rise}})$, which only has to be calculated once a day. Therefore, each prediction only requires 4 additions, 7 multiplications, and 1 division.

The number of operations and memory overhead required for the different SAA implementations is shown in Table 2. We assumed that integer and float numbers are stored as 16 and 32 bit values, respectively. All SAA variants require the last measured energy value to be stored. Additionally, the original SAA scheme also needs to store the current day of year, the sine and cosine values for the latitude angle, the solar altitude angle at the prediction horizon, and, if solar declination angles have been pre-calculated, their corresponding sines and cosines (730 values). In contrast, SAA-Sine requires the sunrise time of the current day, the current day length, and the sine of the relative angle at the future timeslot to be stored. In all cases, the operations involved in the prediction procedure have a low enough complexity so as to be implemented in low-power WSN nodes.

We performed an analogous analysis to obtain the number of operations per prediction and memory overhead for both Pro-Energy and UD-WCMA schemes. Recall that Pro-Energy needs to compute the similarity of the current day with each profile of the pool using Equation (3) and the weighted average energy harvested during the future timeslot of the most similar profiles using Equations (4) and (5). UD-WCMA requires the computation of the similarity of the current day with stored profiles, as Pro-Energy does, as well as the corresponding weighting factors α,β and GAP using Equations (8), (10), and (12), which involves the computation of several standard deviations (see Equations (9) and (13)). The computation of standard deviations requires one to perform several square roots. To avoid any possible bias in favor of our scheme, we assumed that the time complexity for computing a square root was equivalent to that of a multiplication. In addition, both schemes must also update their pool of profiles once a day. Although not considered when estimating the number of operations, recall that both Pro-Energy and UD-WCMA schemes must sort the stored energy profiles by their similarity with the current day, which requires some extra operations for each prediction. Regarding their memory needs, these schemes require the storage of the pool of energy profiles, each one containing the energy harvested during each of the timeslots of a given past day, and the energy harvested during the timeslots of the current day. In view of the results obtained, we can affirm that our proposal is able to provide more accurate predictions in most scenarios using considerably less memory and performing fewer operations.

## 6. Conclusions

This paper presents the SAA (Solar Altitude Angle) predictor, a novel energy prediction model for solar-powered devices. The new technique obtains accurate predictions of future solar energy availability based on the altitude angles of the sun at different times of day. We compared the performance of SAA with some popular techniques using real datasets. Evaluation results show that our proposal is able to provide the most accurate predictions in almost all of the evaluated scenarios by only executing a few low complexity operations and with the lowest memory overhead since, contrary to most state-of-the-art predictors, it does not use past EH patterns to estimate future energy intake.

It is also important to highlight that, unlike most prediction schemes that require the careful tuning of certain configuration parameters to ensure acceptable energy estimations, our energy model provides accurate predictions without requiring any particular configuration for each different scenario. This lack of required settings greatly simplifies the management and setup of the proposed technique in different solar energy harvesters under varying weather conditions and locations.

Further, the SAA predictor does not limit energy estimations to pre-defined instants of time. Although we have presented a new algorithm using equally distributed timeslots to ensure a fair comparison with currently available schemes, our energy model can be used to make predictions for any time of day. Thus, the SAA model can be readily employed in cases where arbitrary prediction horizons are necessary.

## Figures and Tables

**Table 1:** Pro-Energy settings.

Overhead	*D*	*K*	*P*
Low	30	2 (60 min)	1
Medium	60	3 (90 min)	2
High	90	5 (150 min)	5

**Table 2:** Number of operations and memory overhead (in bytes).

	Op. per Day			Op. per Prediction			
Scheme	Add	Mul	Div	Add	Mul	Div	Memory
SAA (stored δ)	1	2	0	9	13	1	2938
SAA	13	19	0	9	13	1	18
SAA-Sine	1	0	1	4	7	1	12
Pro-Low				121	32	0	5952
Pro-Med	Profiles Pool Update			367	65	2	11,712
Pro-High				916	98	5	17,472
UD-WCMA	Profiles Pool Update			109	36	6	1344

## Abbreviations

The following abbreviations are used in this manuscript:

EH	Energy Harvesting
WSN	Wireless Sensor Network
SAA	Solar Altitude Angle
MAE	Mean Absolute Error
MAD	Mean Absolute Deviation
